# Respiratory viral co-infections among SARS-CoV-2 cases confirmed by virome capture sequencing

**DOI:** 10.1038/s41598-021-83642-x

**Published:** 2021-02-16

**Authors:** Ki Wook Kim, Ira W. Deveson, Chi Nam I. Pang, Malinna Yeang, Zin Naing, Thiruni Adikari, Jillian M. Hammond, Igor Stevanovski, Alicia G. Beukers, Andrey Verich, Simon Yin, David McFarlane, Marc R. Wilkins, Sacha Stelzer-Braid, Rowena A. Bull, Maria E. Craig, Sebastiaan J. van Hal, William D. Rawlinson

**Affiliations:** 1 School of Women’s and Children’s Health, Faculty of Medicine, University of New South Wales, Sydney, NSW Australia; 2 Virology Research Laboratory, Serology and Virology Division (SAViD), NSW Health Pathology, Prince of Wales Hospital, Sydney, NSW Australia; 3 Kinghorn Centre for Clinical Genomics, Garvan Institute of Medical Research, Sydney, Australia; 4 St Vincent’s Clinical School, Faculty of Medicine, University of New South Wales, Sydney, NSW Australia; 5 School of Biotechnology and Biomolecular Sciences, Faculty of Science, University of New South Wales, Sydney, NSW Australia; 6 School of Medical Sciences, Faculty of Medicine, University of New South Wales, Sydney, NSW Australia; 7 The Kirby Institute for Infection and Immunity, University of New South Wales, Sydney, NSW Australia; 8 Department of Infectious Diseases and Microbiology, NSW Health Pathology, Royal Prince Alfred Hospital, Sydney, NSW Australia; 9 Research Technology Services, Research Infrastructure Division, University of New South Wales, Sydney, NSW Australia; 10 Institute of Endocrinology and Diabetes, The Children’s Hospital At Westmead, Sydney, NSW Australia; 11 Central Clinical School, University of Sydney, Sydney, NSW Australia

**Keywords:** Viral genetics, Viral infection, SARS-CoV-2, Next-generation sequencing

## Abstract

Accumulating evidence supports the high prevalence of co-infections among Severe Acute Respiratory Syndrome Coronavirus 2 (SARS-CoV-2) patients, and their potential to worsen the clinical outcome of COVID-19. However, there are few data on Southern Hemisphere populations, and most studies to date have investigated a narrow spectrum of viruses using targeted qRT-PCR. Here we assessed respiratory viral co-infections among SARS-CoV-2 patients in Australia, through respiratory virome characterization. Nasopharyngeal swabs of 92 SARS-CoV-2-positive cases were sequenced using pan-viral hybrid-capture and the Twist Respiratory Virus Panel. In total, 8% of cases were co-infected, with rhinovirus (6%) or influenzavirus (2%). Twist capture also achieved near-complete sequencing (> 90% coverage, > tenfold depth) of the SARS-CoV-2 genome in 95% of specimens with Ct < 30. Our results highlight the importance of assessing all pathogens in symptomatic patients, and the dual-functionality of Twist hybrid-capture, for SARS-CoV-2 whole-genome sequencing without amplicon generation and the simultaneous identification of viral co-infections with ease.

## Introduction

Early description of the first 99 COVID-19 cases in Wuhan suggested that co-infections with other respiratory pathogens were rare^1^. However, more recent data from Northern California, USA demonstrate that rates of respiratory co-infections between SARS-CoV-2 and other respiratory viruses can reach up to 21%^2^. Furthermore, a higher prevalence of co-infection is reported among COVID-19 patients with more severe onset of disease^3^ and the deceased^4^, suggesting that co-infections can significantly worsen the clinical outcome of COVID-19. Despite this evidence supporting the high prevalence of co-infections among SARS-CoV-2 cases and its potentially substantial clinical impacts on COVID-19, existing data on co-infection remain limited by the low representation of the global population and the small number of viruses examined. To date, most studies have tested only a narrow spectrum of viruses using targeted qRT-PCR assays^5,6^, and only one reported on the co-infection rate among SARS-CoV-2 cases in the Southern Hemisphere^7^.

Here we measured the rate of respiratory viral co-infection among SARS-CoV-2 cases in Australia, determined by respiratory virome sequencing (excluding phages) using two commercial hybrid-capture sequencing platforms: (i) Virome Capture Sequencing (VirCapSeq), a collection of ~ 2 million oligonucleotide probes (70–120 mers) targeting all known vertebrate-infecting viruses from Roche Sequencing^8^; and (ii) Twist Respiratory Virus Panel, consisting of 41,047 probes (120 mers) targeting 29 human respiratory viruses representing six major pathogenic viral clades, from Twist Biosciences. Furthermore, we evaluated the feasibility of simultaneously performing SARS-CoV-2 whole genome sequencing (WGS) analysis using data generated from both methods. This demonstrated the utility of such a process, and greater WGS coverage achieved using hybrid-capture sequencing over existing amplicon-based procedures in situations where primer binding sites are abolished by genomic deletions.

## Results

### Rate of viral co-infection in Australian cases

We examined the respiratory virome of 92 SARS-CoV-2 cases who tested positive for SARS-CoV-2 RNA between March and May 2020 in New South Wales (NSW), Australia (Supplementary Table 1). The abundance of SARS-CoV-2 RNA in the respiratory specimens obtained from these cases was diverse, with qRT-PCR cycle threshold (Ct) values ranging between 13.3 and 39.7. This was equivalent to a viral load range between 1.4 × 10^8^ copies/mL and less than 10 copies/mL (Supplementary Table 2). Sequencing all vertebrate-infectious viruses in these specimens using VirCapSeq hybrid-capture generated a total of 982 million raw reads, an average of 3.6 million adapter/host filtered reads per sample.

**Figure 1:**
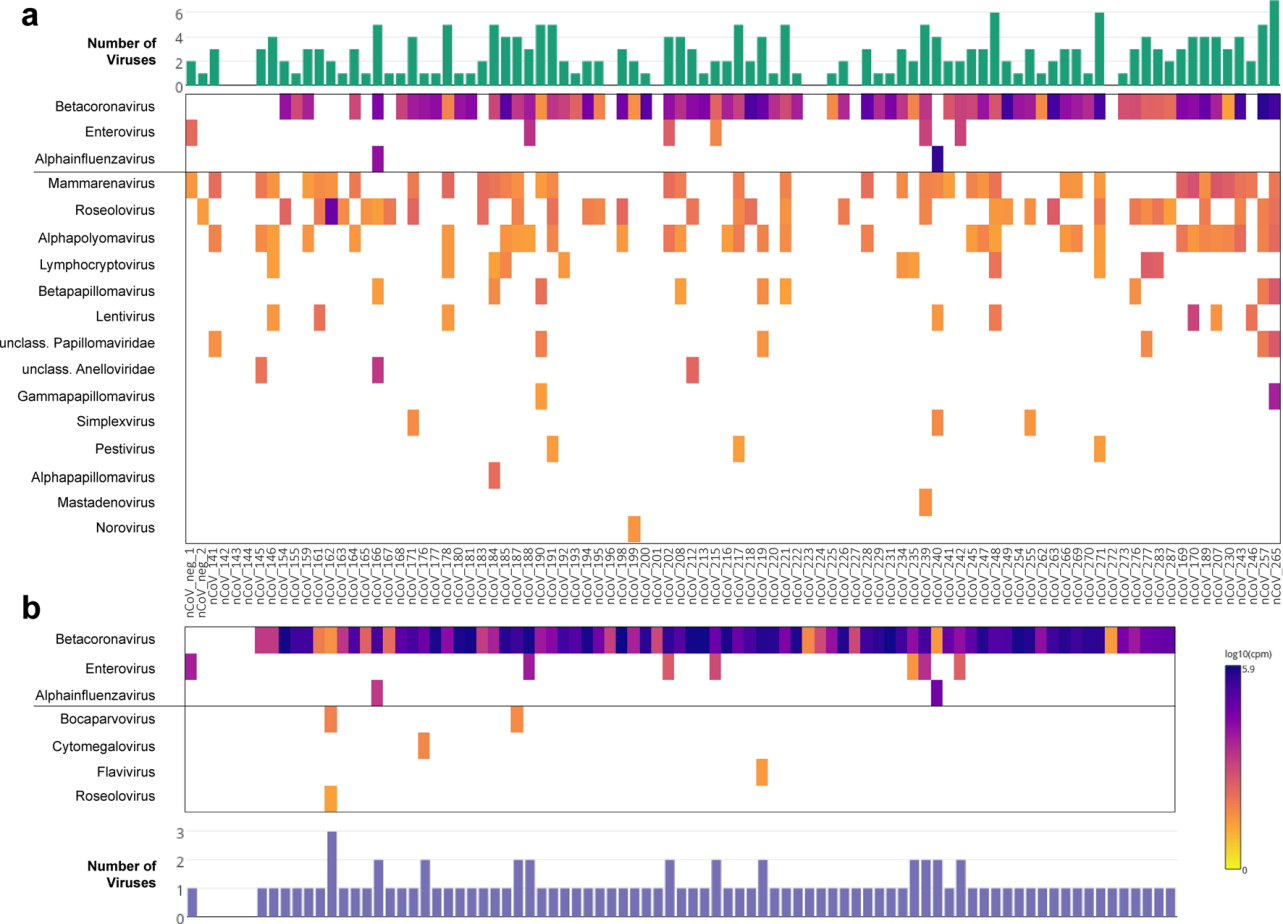
Viruses detected in SARS-CoV-2 case specimens by two hybrid-capture sequencing approaches. (**a**) VirCapSeq (n = 92); and (**b**) Twist Respiratory Virus Panel (n = 83). Heatmap of viral reads in log scale and represented at the genus level. Sample IDs apply for both panels (**a**) and (**b**), indicating overlapping samples sequenced by both approaches. Horizontal line separates respiratory viruses (above) from non-respiratory viruses (below). Bar charts indicate number of viruses detected per specimen. *nCoV_neg_1 & 2* are clinical control specimens from two individuals confirmed negative for SARS-CoV-2 by qRT-PCR.

Overall, 47 species of viruses belonging to 17 different genera were detected with a minimum of 20 virus-classified reads per million (rpM; Supplementary File 1; Fig. 1a). None of these viruses were detected in the negative controls (SSC_1 and SSC_2), ruling out laboratory contamination or index switching as a source of spurious virus detection (Fig. S1a). SARS-CoV-2 reads above the positivity threshold were detected in 80% of samples (74/92), of which the highest Ct was 39.7. No SARS-CoV-2 reads were detected in the coronavirus-negative clinical controls (nCoV_neg_1 and _2; Fig. 1a). Among other respiratory viruses, picornaviruses (all of which were rhinoviruses) were detected in 5% (5/92) of cases and in nCoV_neg_1, and influenzavirus A in 2% (2/92) of cases. Consistent with our published data in other cohorts^9–11^, the non-respiratory viruses detected included mammarenaviruses (41%), roseoloviruses (36%), alphapolyomaviruses (35%), papillomaviruses (20%), lymphocryptoviruses (12%), lentivruses (9%), anelloviruses (3%), simplexviruses (3%), pestiviruses (3%), mastadenovirus (1%) and norovirus (1%). Overall, sequences of viruses other than SARS-CoV-2 were detected in 74% (68/92) of cases, and the rate of co-infection between SARS-CoV-2 and other respiratory viruses was 8% (7/92).

### Validation using the Twist Respiratory Virus Panel

The VirCapSeq results were validated in 85 of the same pre-capture libraries, characterized in parallel using the Twist Respiratory Virus Panel that targets 29 human respiratory viruses. In total, 747 million raw reads were generated, averaging 7.4 million filtered reads per sample. Overall, 20 species of virus were detected, belonging to seven genera (Supplementary File 1; Fig. 1b). None of these viruses were detected in the negative controls (SSC_3 and SSC_4), ruling out laboratory contamination or index switching as a source of spurious virus detection (Fig. S1b). Some sequences in the negative controls were determined as false-positive hits to human adenovirus B, arising from alignment of reads to human genomic DNA sequences cloned within an adenovirus vector backbone (Fig. S1c). SARS-CoV-2 reads were detected in 95% (79/83) of cases, including 16/83 samples undetected using VirCapSeq. SARS-CoV-2 was absent in both coronavirus-negative controls. Consistent with VirCapSeq results, rhinovirus and influenzavirus were the only other respiratory viruses detected. Moreover, the Twist and VirCapSeq panels showed good concordance, with all samples positively identified for these viruses being detected on both platforms, with the exception of one additional rhinovirus-positive case detected by Twist (nCoV_235; Fig. 1a,b). We note that this discordant case was only marginally above the positivity threshold and reads covered < 5% of the reference genome (Supplementary Table 3). Other low-level positives included non-respiratory viruses: bocaparvoviruses, cytomegalovirus, flavivirus and roseolovirus (Fig. 1b).

### Validation across multiple bioinformatics pipelines

The choice of de novo assembler can profoundly impact the analysis and interpretation of virome sequencing data^12^. To test whether the respiratory viral co-infections detected by VirCapSeq and Twist capture sequencing were reproducible using other pipelines that apply a different assembler or a k-mer approach, we compared IDseq results to outputs of VirMAP^13^ and OneCodex^14^ (Supplementary Table 4), respectively. Although the total number of taxonomically classified reads varied across the three pipelines for all respiratory viruses, IDseq and VirMAP were fully concordant for rhinovirus and influenzavirus positive samples. There were clear differences in the number samples identified as SARS-CoV-2 positive, OneCodex identifying the fewest despite reporting the highest total number of SARS-CoV-2 classified reads. IDseq was the most sensitive in identifying SARS-CoV-2 positive samples.

### Complete genome coverage of co-infecting influenzavirus

**Figure 2:**
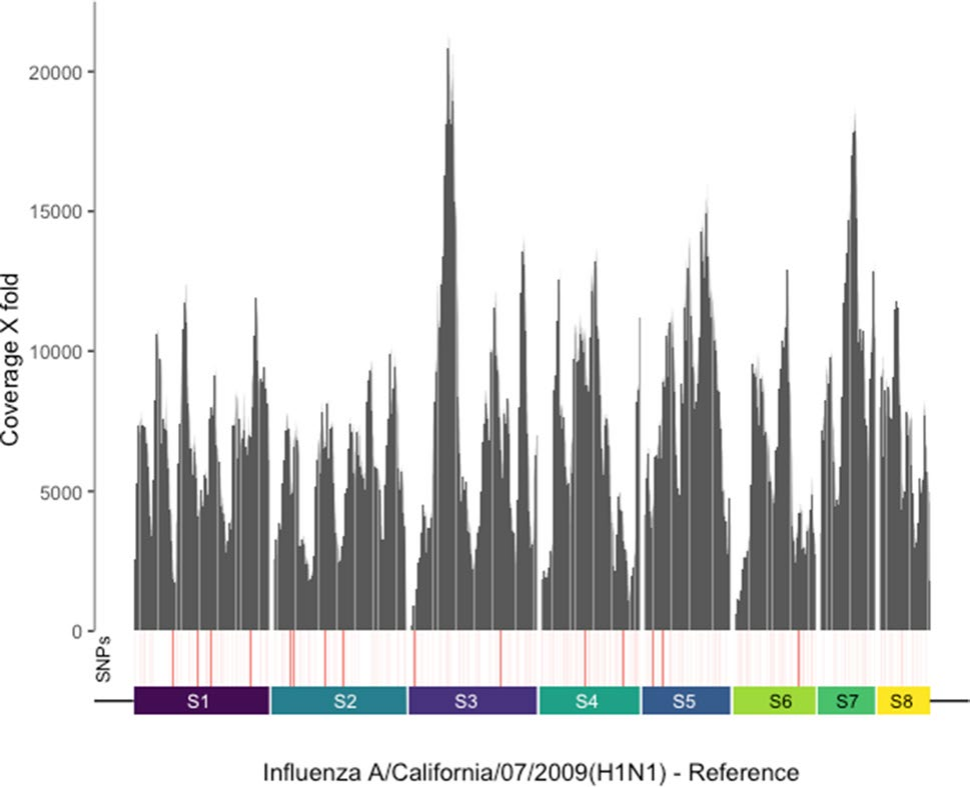
Full genome coverage of co-infecting influenzavirus. Coverage plot of sequence reads generated by Twist capture sequencing of the SARS-CoV-2 case specimen nCoV_240, aligned to the influenzavirus A reference genome across eight different segments (S1-S8). Depth represented as X fold coverage. Single nucleotide polymorphisms (SNPs) detected at positions across the genome are indicated in red, greater intensity of red indicates higher % frequency.

Unlike targeted qRT-PCR assays, virome capture sequencing can be used to determine the genome sequences of coinfecting viruses, informing investigations of virus transmission and evolution. We assessed the suitability of both panels for genome sequencing of coinfecting respiratory viruses. No clear difference was observed between VirCapSeq and Twist capture with respect to rhinovirus genome coverage. Breadth of coverage ranged between 24.3–97.4% and 14.7–75.2% of the reference genome, respectively in samples positive for rhinovirus on both platforms (Supplementary Table 3; Fig. S2). In contrast, VirCapSeq consistently achieved higher mean depth of coverage across most segments of the influenzavirus (Supplementary Table 5; Fig. S2). Nevertheless, Twist capture sequencing provided complete genome coverage of co-infecting influenzaviruses at high depth, sufficient to detect single nucleotide variants (SNVs) at the consensus level (Fig. 2). For both rhinoviruses and influenzaviruses, identified types were concordant between VirCapSeq and Twist panels. Therefore, both platforms were suitable for sequence determination of co-infecting respiratory viruses.

### SARS-CoV-2 genome coverage

**Figure 3:**
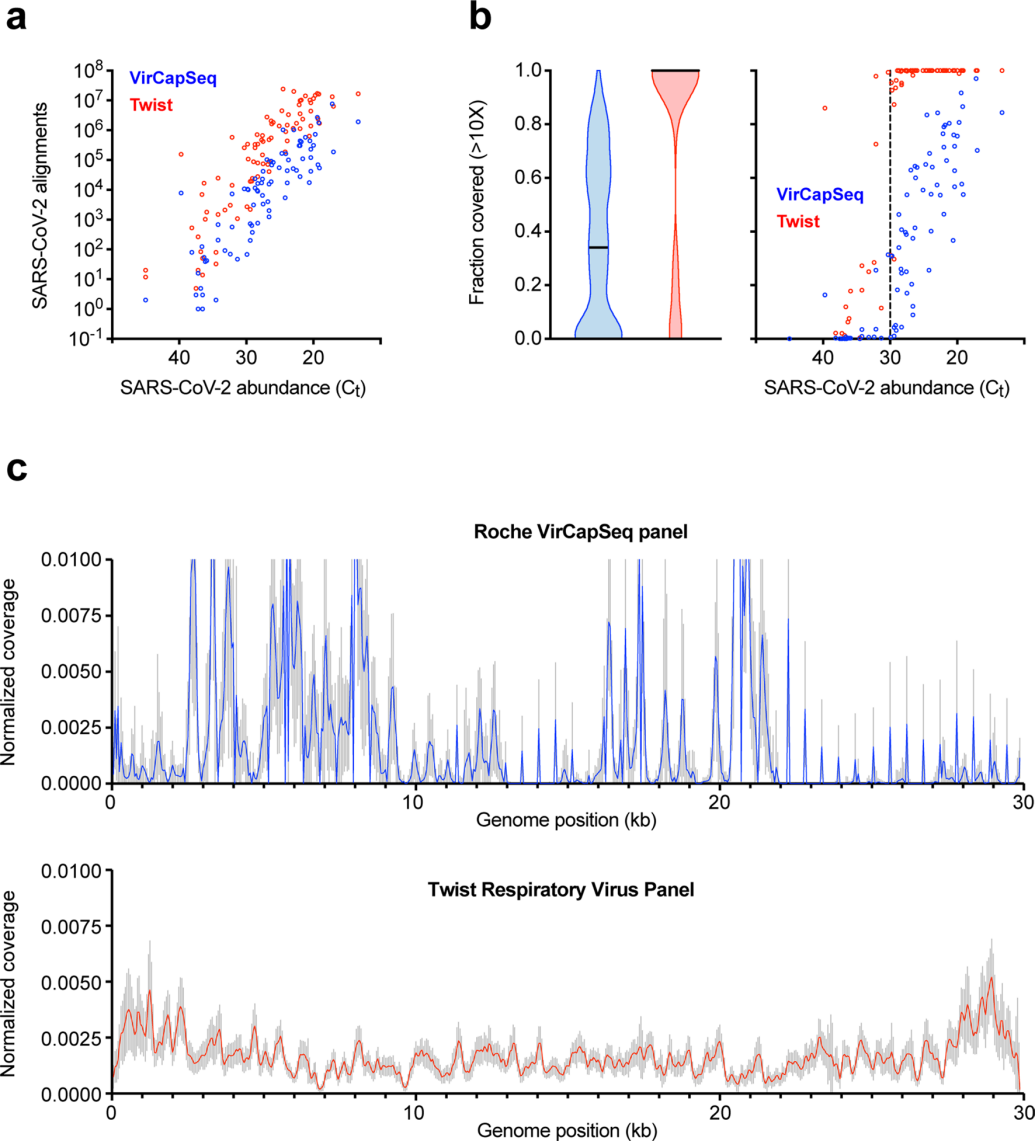
Complete SARS-CoV-2 genome coverage by sequences generated using the Twist Respiratory Virus Panel. (**a**) The number of sequence reads generated by VirCapSeq (blue) and Twist (red) hybrid-capture sequencing, aligned to SARS-CoV-2 in case samples (n = 83) with varying viral load determined by the qRT-PCR cycle threshold (Ct) value. (**b**) The fraction of SARS-CoV-2 genome covered at > 10X depth. Violin plot (left) shows the distribution of genome coverage in samples sequenced by VirCapSeq (blue) and Twist (red) capture with horizontal line indicating the median fraction of genome covered. Vertical dotted line indicates the Ct 30 border (right). (**c**) Distribution of aligned sequence reads across the SARS-CoV-2 reference genome (MN908947.3) and depth of coverage at each position in the genome (50-bp windows) normalized to the average coverage across the whole genome for a given sample.

Viral WGS is being widely applied to study the transmission and evolution of SARS-CoV-2. Amplicon-based sequencing is currently used most frequently but has some limitations in scalability and reproducibility. Given the high sensitivity of the Twist panel for detecting SARS-CoV-2 reads even at viral loads near the qRT-PCR limit of detection (Fig. 3a), we investigated whether the Twist sequencing data provided sufficient coverage of SARS-CoV-2 for WGS analysis. The mean number of SARS-CoV-2 reads detected by the Twist was > tenfold higher than for VirCapSeq (Fig. S3). For samples quantitated with a Ct < 30 on qRT-PCR, Twist capture sequencing achieved a minimum > tenfold sequencing depth across > 90% the SARS-CoV-2 genome for 95% (57/60) of samples (Fig. 3b), and of > 30-fold depth for 89% (53/60) of samples (Fig. S4). The highest Ct at which Twist provided > 90% coverage of > tenfold depth was 32.1. Even in the sample with the lowest viral load (Ct 39.7), 91% of genome was covered at 1X depth (Supplementary File 2).

Compared with the Twist platform, VirCapSeq did not achieve > 90% coverage even at 1X depth for all samples, except two with Ct < 20 (Fig. 3b; Fig. S4; Supplementary File 2). To test whether this low genome coverage was primarily due to the fewer number of SARS-CoV-2 reads detected compared to Twist, SARS-CoV-2 reads in the Twist dataset were sub-sampled to equal to that of VirCapSeq. Even after sub-sampling, Twist-enriched sequences maintained > 90% coverage of 10X depth across the SARS-CoV-2 genome in > 50% of the samples (Fig. S5). This demonstrated that the superior coverage of the SARS-CoV-2 genome achieved using Twist capture sequencing was only partially due to the higher number of SARS-CoV-2 reads. Coverage heterogeneity was the more important determinant, with reads being uniformly distributed across the SARS-CoV-2 genome in Twist samples but scattered unevenly for VirCapSeq (Fig. 3c). Therefore, while the VirCapSeq panel is suitable for detection of SARS-CoV-2 and coinfecting viruses, it is unsuitable for SARS-CoV-2 WGS.

### Detection of inter-individual variation of SARS-CoV-2

We evaluated the capacity to identify inter-individual genetic variation of SARS-CoV-2 from Twist sequencing data. Among 83 cases examined by Twist capture sequencing, the SARS-CoV-2 genomes of 48 cases were previously characterized from same samples through amplicon-based WGS on the Illumina platform. This confirmed the presence of inter-individual single nucleotide variants (SNVs) at the consensus level^15^. Taking the amplicon-WGS data as the truth set, we assessed the sensitivity and precision of consensus sequence variants detected from Twist sequences. Overall, 338 consensus level SNVs were detected with 96% sensitivity and 98% precision, perfectly identified in 88% (42/48) of samples examined (Supplementary Table 6).

### Detection of ORF8 deletion and validation using amplicon WGS

**Figure 4:**
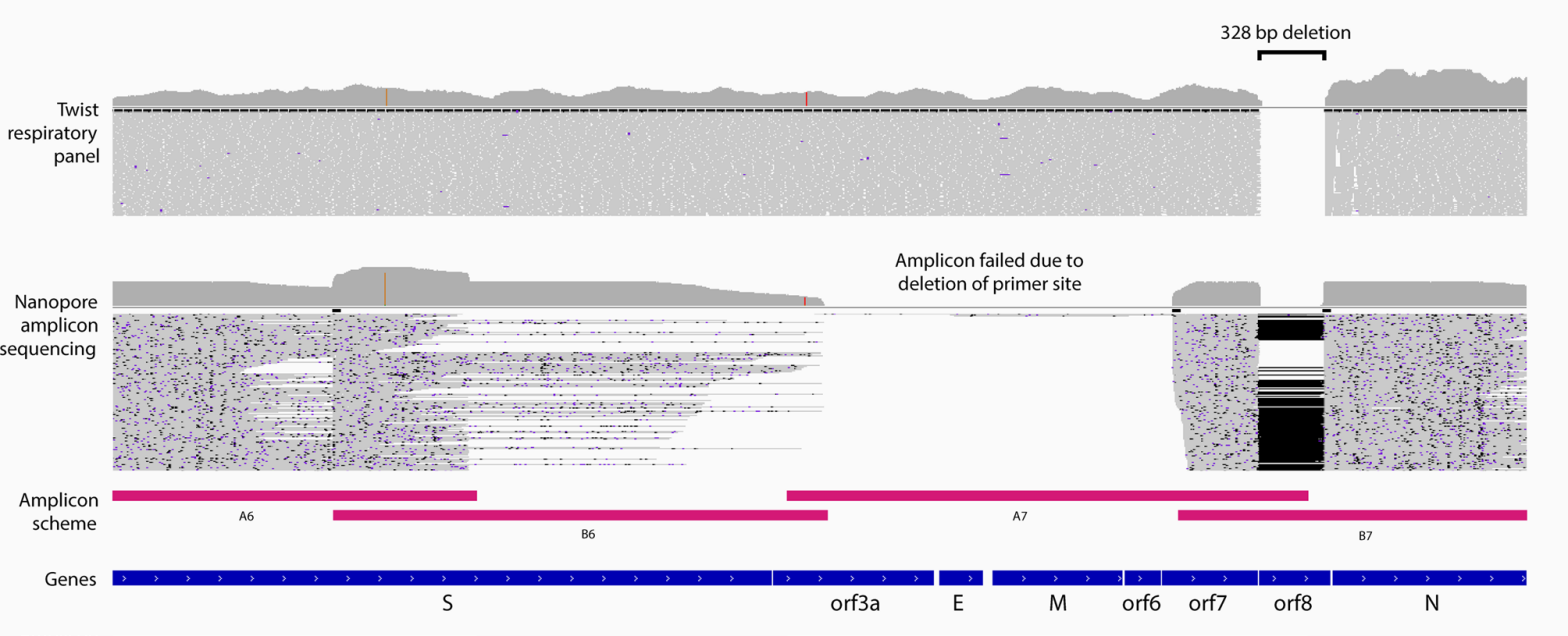
Confirmation of a 328 nt *ORF8* deletion in the SARS-CoV-2. Genome browser view of Twist enriched Illumina (upper) and amplicon-based WGS ONT (lower) sequencing reads aligned across the SARS-CoV-2 genome of nCoV_200 case specimen, zoomed in at the site of 328 nt *ORF8* deletion. ONT sequence alignment shows loss of coverage in the region targeted by the A7 amplicon primers, due to the deletion of a primer-binding site within *ORF8*. In contrast, Twist sequence reads align continuously across this region (Genes *orf3a* to *orf7*)*,* providing greater breadth of coverage of the reference genome (MN908947.3).

Multiple studies report major structural variations (SVs) in the SARS-CoV-2 genome^16–19^, namely the 382 nt deletion in the *open reading frame 8* (*ORF8*), associated with changes in the replicative fitness^17^ and milder infections^18^. Therefore, we investigated if similar *ORF8* deletions could be detected from the cases examined by Twist capture sequencing. We identified two cases with a common 328 bp deletion in *ORF8* (nCoV_200 and nCoV_225). Providing further validation, the same deletion was detected in both cases through amplicon based WGS (Fig. 4; Fig. S6), using Oxford Nanopore Technology (ONT). Interestingly, in both cases, the deletion abolished a primer site, causing the failure of an adjacent amplicon (2.5 kb) and resulting in incomplete coverage of the SARS-CoV-2 genome when profiled by amplicon sequencing. By contrast, hybrid capture sequencing was able to achieve complete genome coverage. This demonstrates that Twist capture sequencing achieves sufficient coverage to reliably detect large deletions in the SARS-CoV-2 genome for clinical specimens of Ct < 30 and is more robust to large deletion or rearrangements in the genome, which can disrupt amplicon schemes.

## Discussion

Determining the co-infection rate and consequent clinical impacts on COVID-19 is critical, particularly where therapeutic interventions for some coinfecting agents such as influenzavirus are available. In this study, we sequenced the respiratory virome using two hybrid-capture approaches and multiple taxonomic read classification pipelines, demonstrating an 8% rate of co-infection with other respiratory viruses among SARS-CoV-2 cases in Australia. This is less than half the rate reported in Northern California^2^, but greater than the initial estimates from Wuhan (0.0%)^1^ and rates of viral co-infection reported from Chicago (1.6%)^20^, New York (2.0%)^21^, Singapore (1.4%)^22^, Barcelona (0.6%)^23^ and Turkey (2.0%)^6^. Furthermore, it is higher than the 4.6% of co-infection observed among 175 cases from the same region, diagnosed and tested during a similar time period using multiplex qRT-PCR^7^. Such high inter- and intra-regional variability warrants further investigation, particularly in developed countries with similar SARS-CoV-2 incidence to Australia. Recent data from Iran^4^ and Poland^24^ support higher mortality of COVID-19 patients as a result of respiratory viral co-infections.

Previous studies have reported co-infections between SARS-CoV-2 and common respiratory viruses including rhinovirus, influenzavirus, meatapneumovirus, parainfluenzavirus and respiratory syncytial virus^5,25^. In our results, co-infections between SARS-CoV-2 and rhinoviruses (6%) were predominant, lower for influenzaviruses (2%). Interestingly, co-infection between SARS-CoV-2 and influenza was not observed in a recent report of Australian SARS-CoV-2 cases^7^. The case specimens examined in the present study were collected between March and May 2020, overlapping with the start of the Southern Hemisphere influenza season. In Australia, the flu season thus far has reported > 90% reduction in incidence of influenzavirus infections compared to the same period in 2019 as a result of social distancing and mandatory quarantine measures applied during the COVID-19 pandemic^26^. Therefore, the significant reduction in circulating influenzavirus may have contributed to its low co-infection rate with SARS-CoV-2.

Our study has several limitations. All specimens examined in this study were freeze-thawed twice before library preparation. This may have prevented detection of viruses that were originally at very low titer. Therefore, the actual rate of co-infection may exceed 8%. To eliminate this potential in future analyses, double-stranded cDNA should be generated on the same day as SARS-CoV-2 qRT-PCR, from the same nucleic acid extracts avoiding freeze–thaw. A key limitation of our analysis was the lack of clinical metadata, precluding examination of potential associations between respiratory viral co-infection with SARS-CoV-2 and clinical outcomes of COVID-19. Although comparable to other co-infection studies to date, our sample size was small and included only a single timepoint for each case. Nevertheless, this represents the largest metagenomic sequencing study to date, examining co-infections between SARS-CoV-2 and other respiratory viruses.

There is growing appreciation for SARS-CoV-2 WGS as an essential tool to investigate the transmission and evolution of SARS-CoV-2, critical for research and public health responses to COVID-19^15–17,27–31^. Existing WGS approaches can be divided into two main categories: 1. Amplicon sequencing; and 2. Hybrid-capture sequencing using SARS-CoV-2-specific probes. Neither are capable of simultaneously detecting co-infecting viruses. Our analysis of the SARS-CoV-2 genome using Twist-enriched sequenced demonstrated high breadth and depth of coverage for samples with Ct < 30, sufficient for downstream analysis of SNV, indels and SVs. This was despite using single-end sequence data. Hence, even greater confidence in variant calling can be achieved using paired-end sequencing. Overall, target enrichment sequencing using the Twist Respiratory Virus Panel offers dual-functionality, providing effective characterization of co-infecting respiratory viruses and the full genome of the SARS-CoV-2, simultaneously.

Unlike amplicon sequencing, Twist hybrid-capture does not require generation of SARS-CoV-2 amplicons. This significantly reduces processing time and manual handling, lowering the risk of cross contamination. Using the Twist’s fast hybridization and multiplexed capture workflow, libraries ready for high throughput sequencing can be constructed from clinical specimen extracts in < 8 h. Although the amplicon approach can also construct libraries within a similar timeframe, in our experience of using two published amplicon WGS methods^15,28,32^, generating amplicons often took longer than anticipated due to certain parts of the genome amplifying poorly, requiring continuous optimization. Furthermore, in this study, all libraries were hybridized with Twist probes for 2 h. However, this can be reduced to 30 min with minimal loss in capture efficiency.

The default protocol for Twist hybrid-capture supports multiplexing up to 8 libraries (8-plex) per capture hybridization, combining libraries by equal mass to make up 1.5 µg of total DNA, or up to 4 µg total without compromising the efficacy of target enrichment. In this study, we performed Twist capture on libraries pooled up to 20-plex, whilst still maintaining the 4 µg total DNA limit. This highly multiplexed sample processing significantly reduced processing time, labor and cost per sample. Current per-sample cost of Twist Respiratory Virus Panel in a 20-plex sample format is $25 USD. This compares favorably with the cost of VirCapSeq (~ $23 USD per sample), particularly given its advantages in sensitivity and genome coverage of SARS-CoV-2.

Taken together, we provide a practical and cost-effective strategy for characterizing both respiratory viral co-infections and the full SARS-CoV-2 genome simultaneously, from clinical specimens with Ct < 30 or viral load > 3,000 copies/mL. We also recommend IDseq as the preferred pipeline for taxonomic classification of viral sequences in SARS-CoV-2 specimens, based on its high sensitivity for SARS-CoV-2 and other respiratory viruses, ease of use, and minimal requirements in terms of infrastructure and bioinformatic expertise. We envision broad application of our approach across research and clinical settings.

## Methods

### Clinical samples and SARS-CoV-2 qRT-PCR

Respiratory specimens of SARS-CoV-2 cases (adults) in NSW diagnosed between March and May 2020 were obtained from at the Prince of Wales Hospital in Randwick, Sydney, Australia. Ethical approval and informed consent waiver was received from the South Eastern Sydney Local Health District Human Research Ethics Committee (2020/ETH02639). All methods were performed in accordance with the relevant guidelines and regulations. Prior to this study, samples were freeze-thawed twice and stored at − 80 °C following diagnostic testing at the NSW Health Pathology East Serology and Virology Division (SaViD). In total, 92 nasopharyngeal swabs suspended in Viral Transport Media (VTM) were selected for virome capture sequencing, all positive for a combination of four SARS-CoV-2 target genes (RdRp, S, N and E) on the Allplex SARS-CoV-2 qRT-PCR Assay (Seegene, Seoul, Korea). The approximate copy number of SARS-CoV-2 RNA was calculated by plotting the Ct against a standard curve built from tenfold serial dilution of a quantified N-gene plasmid control, developed inhouse. To use as controls, two nasopharyngeal swabs confirmed negative for SARS-CoV-2 from the same diagnostic laboratory and two negative controls prepared from purified Salmon Sperm DNA (15632-011; Thermo Fisher Scientific, MA, USA) were also sequenced.

### Total nucleic acid extraction, cDNA synthesis and library prep

Total nucleic acid was extracted from 200 µL of swab suspension in VTM, using the AllPrep PowerViral DNA/RNA kits (Qiagen, Hilden, Germany) with bead-beating and phenol/chloroform, following manufacturer’s protocol. Using Superscript III (Thermo Fisher Scientific) and Klenow Fragment (NEB, MA, USA) with random hexamers, the RNA portion was converted into double-stranded cDNA. Illumina sequencing libraries were prepared from 1ug of double-stranded DNA/cDNA mixture, using the KAPA Hyper Plus (Roche, Basel, Switzerland) kit with Unique Dual-Index adapters. Libraries were quantified by picogreen (Thermo Fisher Scientific) and the size distribution of library fragments were measured on the LabChip GX Touch 24 (Perkin Elmer, MA, USA) bioanalyzer.

### Target enrichment sequencing

For VirCapSeq hybrid-capture, individual libraries (92 cases, 2 coronavirus-negative controls and 2 salmon sperm DNA controls) were combined by equal mass into two capture pools (48-plex) and hybridized to probes (VirCapSeq-VERT design; Roche) for 16 h as previously described^11^, following the SeqCap EZ HyperCap Worklfow v2.3 (Roche). For hybrid-capture using the Twist Respiratory Virus Research Panel (103067; Twist Biosciences, San Francisco, CA), 87 libraries (83 cases, 2 coronavirus-negative controls and 2 salmon sperm DNA controls) were combined by equal mass into five capture pools (16- to 20-plex; average 17-plex). Pools were hybridized to probes for 2 h, following the Fast Hybridization Workflow (Twist Biosciences). VirCapSeq and Twist post-capture library pools were PCR amplified 16 cycles and single-end sequenced (1 × 100 bp) separately, up to 96 barcoded libraries maximum per lane of a NovaSeq 6000 S1 flowcell (Illumina, San Diego, CA) at the Ramaciotti Centre for Genomics (UNSW Sydney, Australia).

### Taxonomic classification of metagenomic reads

By default, taxonomic classification of viral reads in all samples was achieved using *IDseq* (v4.0)^33^, a cloud-based, open-source bioinformatics pipeline for metagenomic sequencing data. Raw fastq files were uploaded to the IDseq portal (https://idseq.net) using the Amazon Web Services (AWS) Command Line Interface. All IDseq scripts and user instructions are available at https://github.com/chanzuckerberg/idseq-dag. In brief, adapter and human host sequences were filtered, and remaining short-read sequences were aligned to the NCBI nucleotide (nt) and nonredundant protein (nr) databases (ftp://ftp.ncbi.nlm.nih.gov/blast/db/FASTA/) using *GSNAPL*^34^ and *RAPsearch2*^35^, respectively. Putative accessions were assigned to each read using the NCBI *accession2taxid* database (ftp://ftp.ncbi.nih.gov/pub/taxonomy/accession2taxid) and a *BLAST* + (v 2.6.0)^36^ database. In parallel, short reads were de novo assembled into contigs using *SPAdes*^37^. Raw reads were mapped back to the resulting contigs using *Bowtie2*^38^, to identify the contig to which they belong. Finally, each contig was aligned to the set of possible accessions represented by the BLAST database, to improve the specificity of alignments to all the underlying reads. Only viruses detected at ≥ 20 rpM based on nt alignments (NT rPM) were deemed positive and included in heatmaps generated using *iheatmapr*^39^.

For the comparative analysis between IDseq and other bioinformatic pipelines, taxonomic read classification summaries were generated using *VirMAP*^13^ and *OneCodex*^14^. *VirMAP* was installed and run on the National Computing Infrastructure (NCI) HPC *Gadi* with modifications described at https://github.com/rsoftone/virmap. *OneCodex* is a premium cloud-based pipeline, for which raw fastq files generated using Twist hybrid-capture sequencing were uploaded and summary reports downloaded using the web browser interface (https://app.onecodex.com/).

### Virus genome assembly, coverage analysis and variant detection

For samples containing sequences corresponding to rhinoviruses/enteroviruses and influenzaviruses, host-filtered sequences from IDseq were mapped to their respective reference genome sequence obtained from the NCBI database using *minimap2* (v2.17-r941)^40^. Coverage statistics and SNV reports were generated from the sorted bam file using *qualimap* (v2.2.2-dev)^41^ and *freebayes* (v1.3.2-dirty)^42^, respectively. Genome assemblies and coverage statistics in were also generated in Geneious Prime (v2020.2.2; Biomatters Ltd.)^43^ for supplementary tables and figures.

For all SARS-CoV-2 genome assemblies, host-filtered reads were aligned to the Wuhan-Hu-1 reference genome (MN908947.3) using *bwa mem* (0.7.12-r1039)^44^, with only MapQ = 60 alignments retained. Per-base coverage was calculated at each position in the SARS-CoV-2 genome using *bedtools coverage* (v2.25.0)^45^. Coverage breadth (fraction of positions covered) was calculated at a range of different minimum depths (≥ 1X, 2X, 5X, 10X, 20X, 30X, 50X, 100X). For samples where SARS-CoV-2 was detected at Ct < 30, SNVs were detected using *samtools mpileup* (v1.9)^46^ and *Varscan2 mpileup2snp* (v2.4.3)^47^. SNVs with a minimum read-count frequency ≥ 80% were retained as consensus SNVs. Variant detection performance was evaluated by comparing detected consensus SNVs in Twist capture samples to matched specimens analyzed elsewhere^15^ by amplicon-based WGS (n = 48). Validation of *ORF8* deletions by amplicon-based WGS using the ONT platform was performed as previously described^15^ and alignment of reads across the deletion site was visualized in Integrative Genomics Viewer (IGV; v2.8)^48^.

## Supplementary Information

Supplementary Information.

Supplementary File 1.

Supplementary File 2.

Supplementary Table 2.

Supplementary Table 6.

## Data Availability

All de-identified metagenomic sequencing data (raw and processed fastq files) will be made publicly available in time for publication.
