# Supplementary material for: Respiratory viral co-infections among SARS-CoV-2 cases confirmed by virome capture sequencing
Source: Sci Rep. 2021 Feb 16;11:3934. doi: 10.1038/s41598-021-83642-x (PMC7887263; doi:10.1038/s41598-021-83642-x)
Supplement: Supplementary file 1 — Supplementary Information. [file 41598_2021_83642_MOESM1_ESM.pdf]

SUPPLEMENTARY INFORMATION

Respiratory viral co-infections among SARS-CoV-2 cases confirmed by virome capture sequencing

Ki Wook Kim[1,2], Ira W. Deveson[3,4], Chi Nam I. Pang[5], Malinna Yeang[2], Zin Naing[2], Thiruni Adikari[6,7], Jillian M. Hammond[3], Igor Stevanovski[3], Alicia G. Beukers[8], Andrey Verich[7], Simon Yin[9], David McFarlane[9], Marc R. Wilkins[5], Sacha Stelzer-Braid[2,6], Rowena A. Bull[6,7], Maria E. Craig[1,2,10], Sebastiaan J. van Hal[8,11] and William D. Rawlinson[1,2,5,6]*

Fig. S1. Viral sequences detected in the negative non-human controls p. 5

Fig. S2. Reference genome coverage of influenzaviruses and rhinoviruses detected. p. 6

Fig. S3. Sensitivity of VirCapSeq and Twist enrichment sequencing in detecting SARS-CoV-2 sequences. p. 7

Fig. S4. SARS-CoV-2 genome coverage at varying levels of depth. p. 8

Fig. S5. SARS-CoV-2 genome coverage following sub-sampling of Twist sequences. p. 8

Fig. S6. *ORF8* deletion detected by Twist enrichment sequencing and amplicon WGS. p. 9

Supplementary Table 1. Characteristics of SARS-CoV-2 cases examined by virome capture sequencing.

Supplementary Table 2. SARS-CoV-2 qPCR cycle threshold values and estimated viral load based on the N-gene standard curve for each specimen.

Table provided as separate Excel file.

Supplementary Table 3. Reference genome coverage statistics for rhinoviruses/enteroviruses detected by target enrichment sequencing using the VirCapSeq and Twist Respiratory Virus capture probes.

Supplementary Table 4. Number of case samples positive for respiratory viruses across three viral sequence classification pipelines based on the 20 viral reads per million raw sequence positivity threshold.

Supplementary Table 5. Reference genome coverage statistics for influenzaviruses detected by target enrichment sequencing using the VirCapSeq and Twist Respiratory Virus capture probes.

Supplementary Table 6. Detection of consensus sequence variants in individual SARS-CoV-2 specimens with Twist enrichment sequencing.

Table provided as separate Excel file.

Supplementary File 1. Viral reads classified at the species and genus level by IDseq from VirCapSeq and Twist hybrid-capture sequencing data.

Taxonomic summaries and read count values provided as an Excel file.

Supplementary File 2. SARS-CoV-2 genome coverage statistics and the respective Ct values for all samples sequenced by both VirCapSeq and Twist hybrid-capture.

Summary of the SARS-CoV-2 aligned reads, % coverage at varying depths and Ct values provided as an Excel file.

Supplementary Table 1. SARS-CoV-2 patient characteristics and sites of specimen collection stratified by non-SARS-CoV-2 respiratory virus status

Characteristic	SARS-CoV-2 positive (n=92)	
	**Positive for other respiratory virus**	**Negative for other respiratory virus**
No. of cases	7	85
No. of samples	7	85
Mean age, y (SD)	57.2 (24.6)	53.5 (19.4)$^{\alpha}$
Female, n (%)	3/7 (43)	38/85 (45)$^{\beta}$
Site of specimen collection, n/total (%)		
Outpatient	7/7 (100)	79/85 (93)
Inpatient	0/7 (0)	6/85 (7)

$^{\alpha}$2-tailed t-test (p=0.636); $^{\beta}$ Pearson's chi-squared test (p=0.925)

Supplementary Table 3. Reference genome coverage statistics for rhinoviruses/enteroviruses detected by target enrichment sequencing using the VirCapSeq and Twist Respiratory Virus capture probes.

Sample	Reference Genome (Genbank Accession)	Reference Sequence Length (nt)	VirCapSeq			Twist Respiratory		
			**No. Reads Aligned**	**% of Reference Sequence**	**Mean depth (X)**	**No. Reads Aligned**	**% of Reference Sequence**	**Mean depth (X)**
nCoV_188	KJ675505.1	7,123	1,149	76.0	16.2	3,311	75.2	46.8
nCoV_202	LC530048.1	7,025	161	46.3	2.4	254	65.2	3.6
nCoV_215	MN306037.1	7,189	52	24.3	0.7	247	74.8	3.5
nCoV_239	FJ445175.1	7,140	604	69.3	8.6	581	15.3	8.2
nCoV_242	MK989738.1	6,866	1,893	97.4	27.8	518	14.7	7.6
nCoV_235	NC_009996.1	7,099	ND	ND	ND	21	4.1	0.3
nCoV_neg_1	KY684757.1	7,206	124	43.7	1.7	1,202	70.4	16.7

ND = Not Detected

Supplementary Table 4. Number of case samples positive for respiratory viruses across three viral sequence classification pipelines based on the 20 viral reads per million raw sequence positivity threshold.

Virus	IDseq		VirMAP		One Codex
	**VirCapSeq (n=92)**	**Twist (n=83)**	**VirCapSeq (n=92)**	**Twist (n=83)**	**Twist (n=83)**
**SARS-CoV-2**					
Positive samples, n (%)	74 (80)	79 (95)	63 (68)	71 (86)	69 (83)
Classified reads, n	32,872,713	224,810,368	2,783,697	40,330,513	664,671,745
**Enterovirus/Rhinovirus**					
Positive samples, n (%)	5 (5)	6 (7)	5 (5)	6 (7)	4 (5)
Classified reads, n	8,066,303	1,110,665	19,200	6,513	2,676
**Influenzavirus**					
Positive samples, n (%)	2 (2)	2 (2)	2 (2)	2 (2)	1 (1)
Classified reads, n	7,440,619	973,417	357,343	236,123	447

Supplementary Table 5. Reference genome coverage statistics for influenzaviruses detected by target enrichment sequencing using the VirCapSeq and Twist Respiratory Virus capture probes.

Sample	Influenzavirus Segment	Reference Genome (NCBI Accession)	Reference Sequence Length (nt)	VirCapSeq			Twist Respiratory		
				No. Reads Aligned	% of Reference Sequence	Mean depth (X)	No. Reads Aligned	% of Reference Sequence	Mean depth (X)
nCoV_166	1	NC_026438.1	2,280	1,579	100.0	68.4	561	100.0	24.3
	2	NC_026435.1	2,274	1,103	98.8	48.5	414	100.0	17.9
	3	NC_026437.1	2,151	1,594	97.4	72.7	484	97.4	21.8
	4	NC_026433.1	1,701	5,514	100.0	41.3	359	100.0	18.7
	5	NC_026436.1	1,497	1,004	99.0	52.5	421	100.0	27.6
	6	NC_026434.1	1,410	736	100.0	46.6	6,045	100.0	109.3
	7	NC_026431.1	982	488	98.7	48.9	332	98.7	32.7
	8	NC_026432.1	863	251	100.0	28.6	229	100.0	23.9
nCoV_240	1	NC_026438.1	2,280	51,756	100.0	2,220.6	9,114	100.0	400.4
	2	NC_026435.1	2,274	48,839	100.0	2,132.4	15,866	100.0	1,318.5
	3	NC_026437.1	2,151	63,628	100.0	2,833.3	8,202	100.0	383.0
	4	NC_026433.1	1,701	70,855	100.0	5,082.6	6,997	100.0	405.6
	5	NC_026436.1	1,497	31,606	100.0	1,735.8	7,289	100.0	488.6
	6	NC_026434.1	1,410	25,404	100.0	1,636.9	5,200	100.0	369.5
	7	NC_026431.1	982	17,433	100.0	1,403.5	7,406	100.0	531.1
	8	NC_026432.1	863	19,242	100.0	2,181.4	26,720	100.0	4,866.8

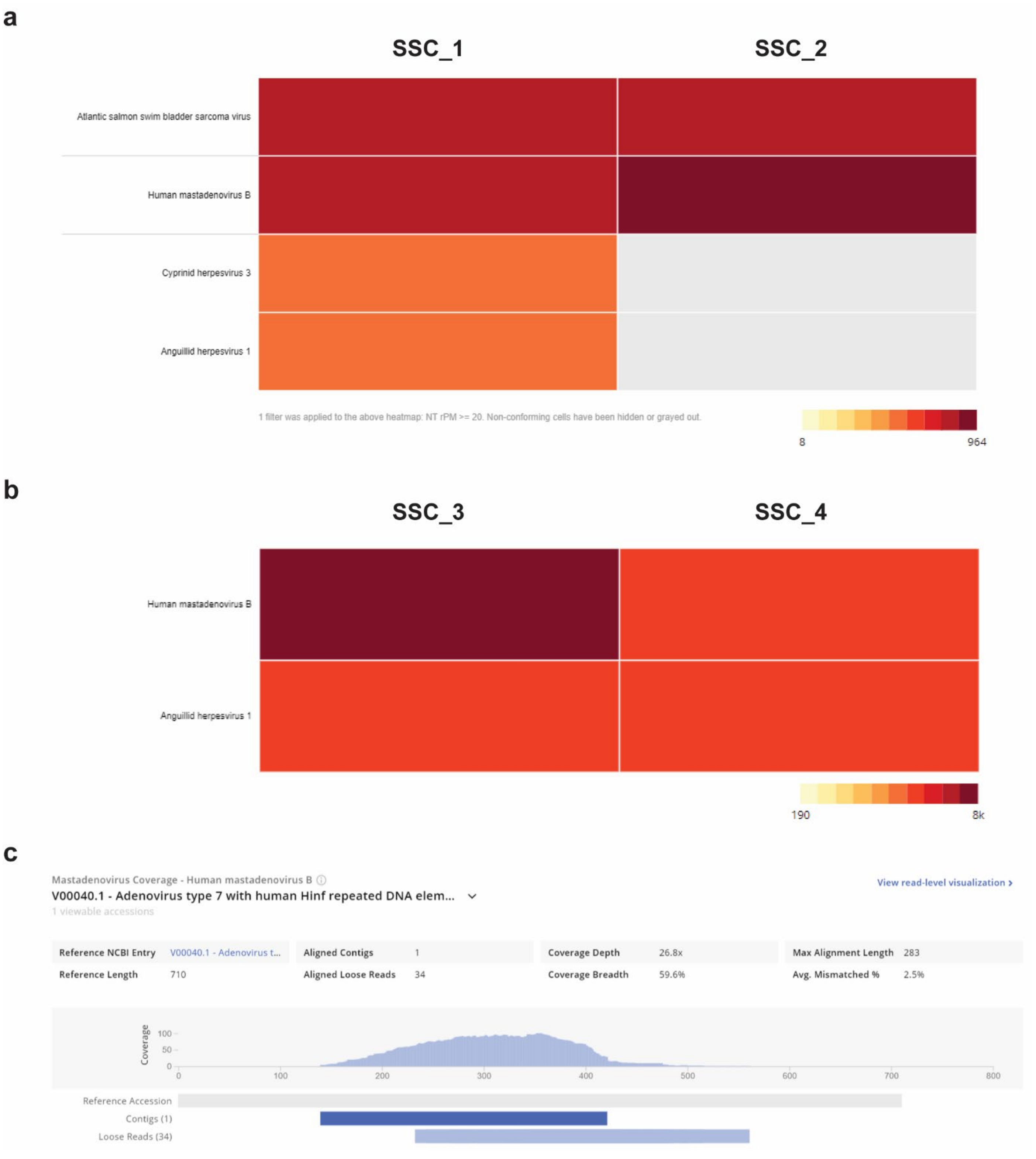

Figure S1. Viral sequences detected in the negative non-human controls. a. Heatmap of viral reads (log scale) detected in Salmon Sperm DNA control (SSC) libraries by VirCapSeq enrichment sequencing (SSC_1 and SSC_2), represented at the genus level. Viral sequences detected for three non-human viruses (Atlantic salmon swim bladder sarcoma virus, cyprinid herpesvirus 3 and anguillid herpesvirus 1) and human mastadenovirus B. **b.** Heatmap of viral reads (log scale) detected in SSC libraries by Twist enrichment sequencing (SSC_3 and SSC_4), represented at the genus level. Viral sequences detected for anguillid herpesvirus 1 and human mastadenovirus B. **c.** Genome assembly (performed within the IDseq online portal) showing alignment of contigs and loose reads from SSC_1-4 to the human Hinf repeated DNA element insert sequence cloned within an adenovirus type 7 backbone (GenBank: V00040.1), confirming false-positivity for human mastadenovirus B.

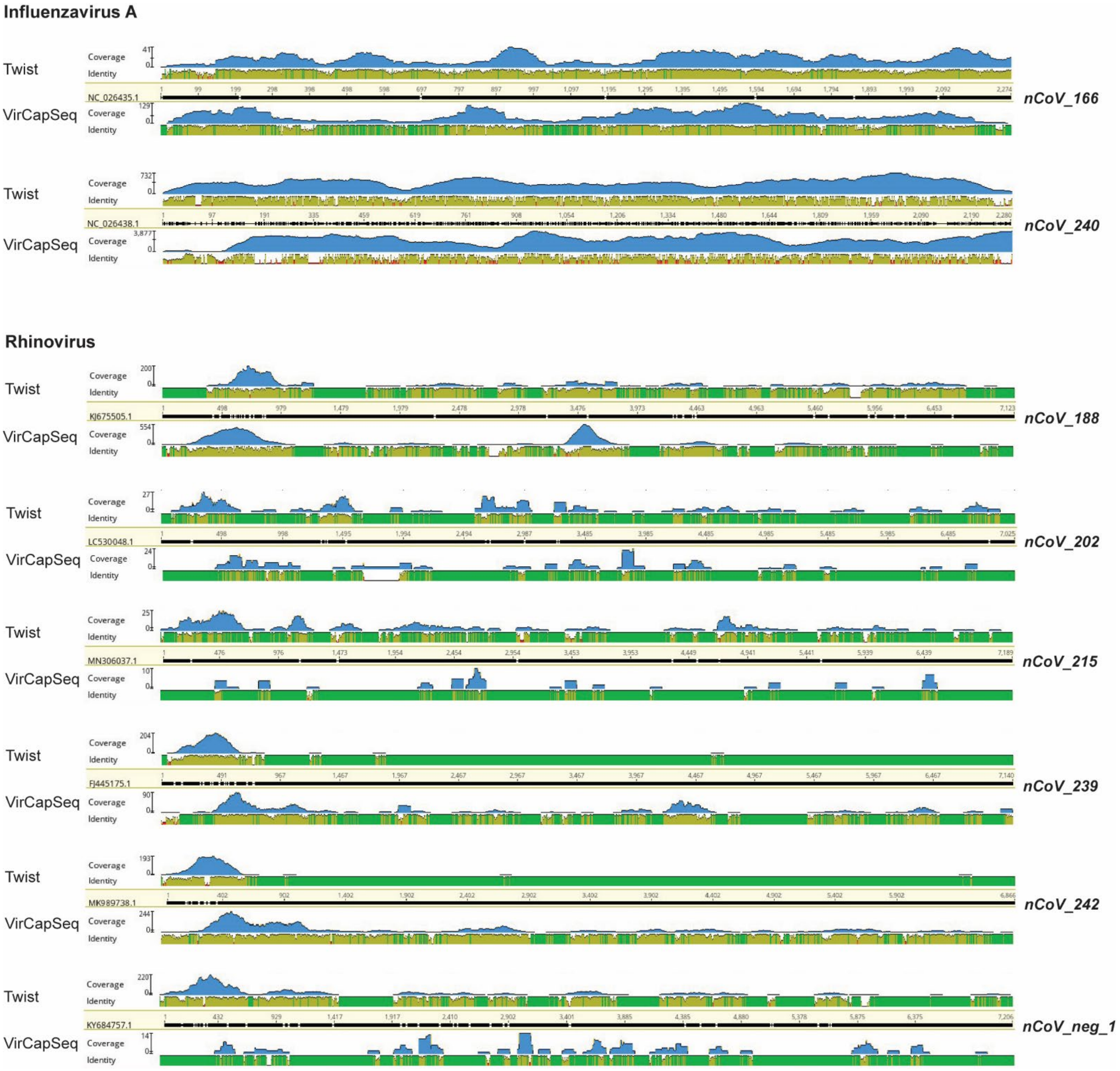

Figure S2. Reference genome coverage of influenzaviruses and rhinoviruses detected. Coverage plots for two samples positive for influenzavirus A (*nCoV_166* and *240*) and six samples positive for rhinovirus (*nCoV_188, 202, 215, 239, 242* and *nCoV_neg_1*) by both VirCapSeq and Twist enrichment sequencing are shown. Depth of coverage (y-axis) plotted against nucleotide position on the reference genome (x-axis), respective Genbank accession shown. Blue peaks indicate coverage at each position and mean pair-wise identity with reference sequence at each position represented (green = 100% identity; greenish-brown = >30%, <100% identity; red=<30% identity).

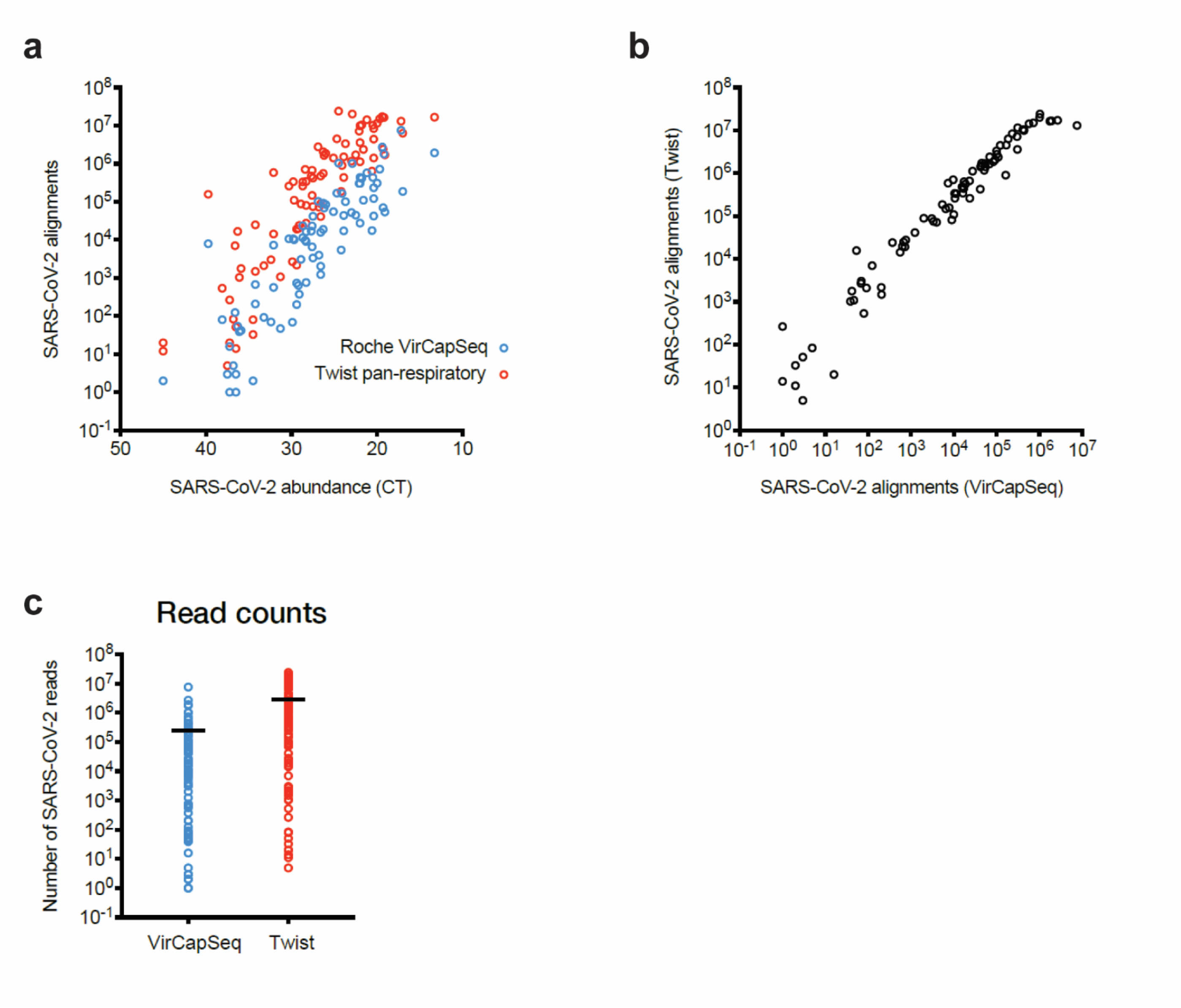

Figure S3. Sensitivity of VirCapSeq and Twist enrichment sequencing in detecting SARS-CoV-2 sequences. a. Number of SARS-CoV-2 aligned reads in each specimen plotted against respective qPCR cycle threshold (Ct) values. **b.** Number of SARS-CoV-2 aligned reads in sequences generated by Twist panel versus VirCapSeq in each specimen. **c.** Number of SARS-CoV-2 reads detected for each specimen using VirCapSeq versus Twist panel. Horizontal line indicates the median.

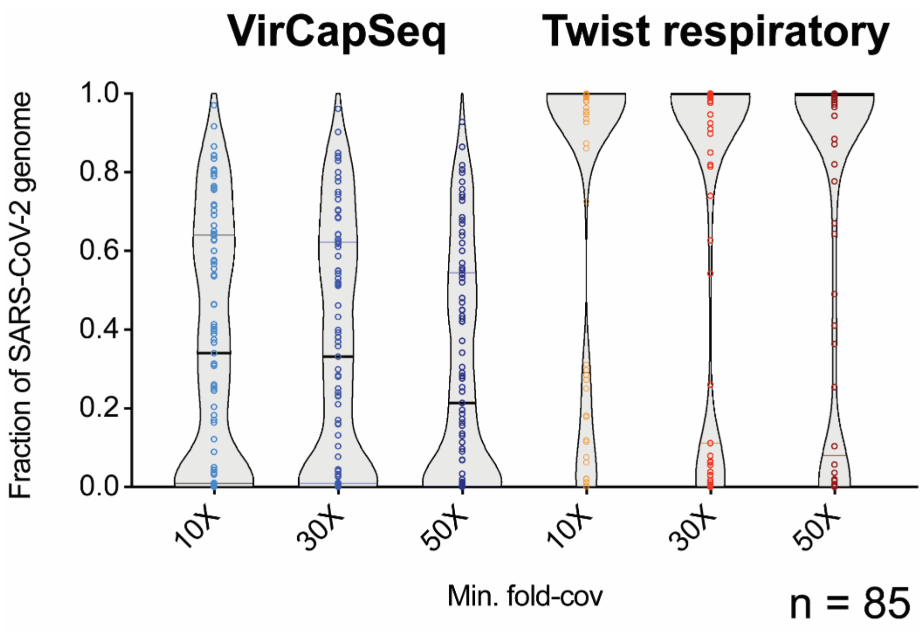

Figure S4. SARS-CoV-2 genome coverage at varying levels of depth. Samples analyzed by both VirCapSeq and Twist enrichment sequencing are represented (n=85). Fraction of SARS-CoV-2 genome covered at varying minimum fold-depth of coverage (10X, 30X and 50X). Horizontal line indicates the median.

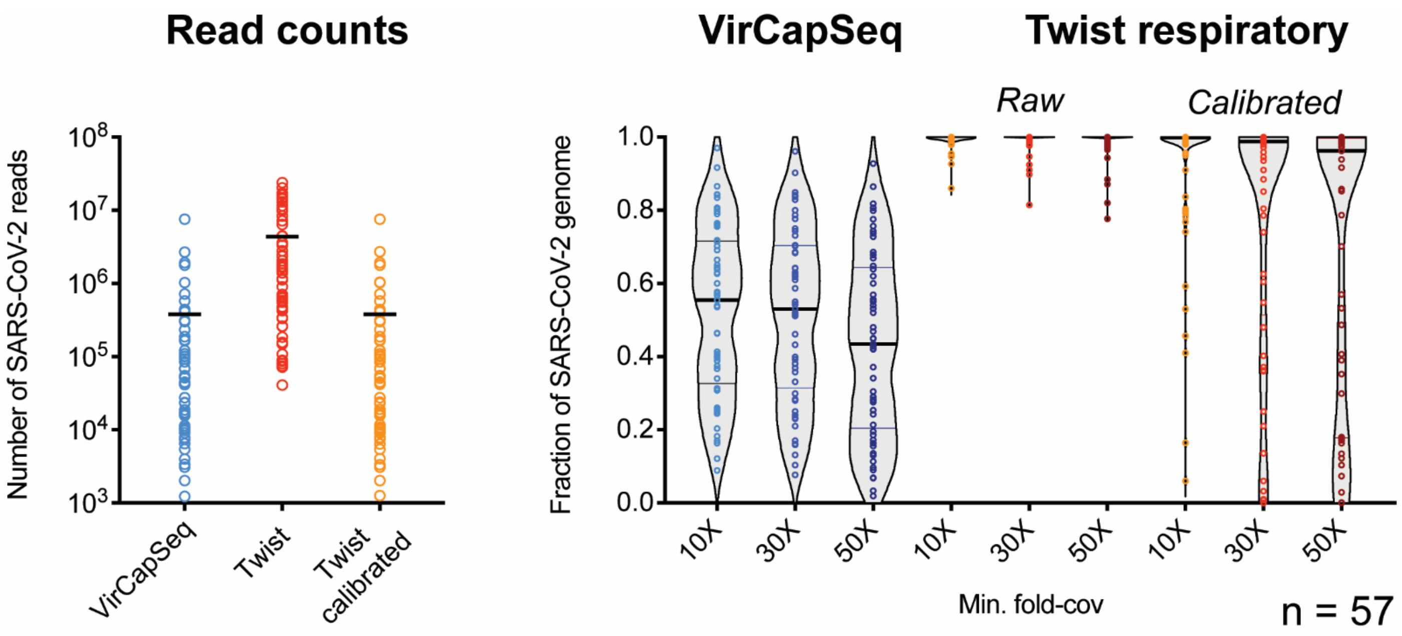

Figure S5. SARS-CoV-2 genome coverage following sub-sampling of Twist sequences. SARS-CoV-2 reads sequenced by Twist-enrichment were sub-sampled for specimens containing $\geq$1,000 SARS-CoV-2 reads (n=57). Total SARS-CoV-2 read counts were sub-sampled to be equal to that of VirCapSeq for each sample (left) and results of the genome coverage analysis at varying depths (10-50X) are shown (right), before ("Raw") and after sub-sampling ("Calibrated"). Horizontal line indicates the median.

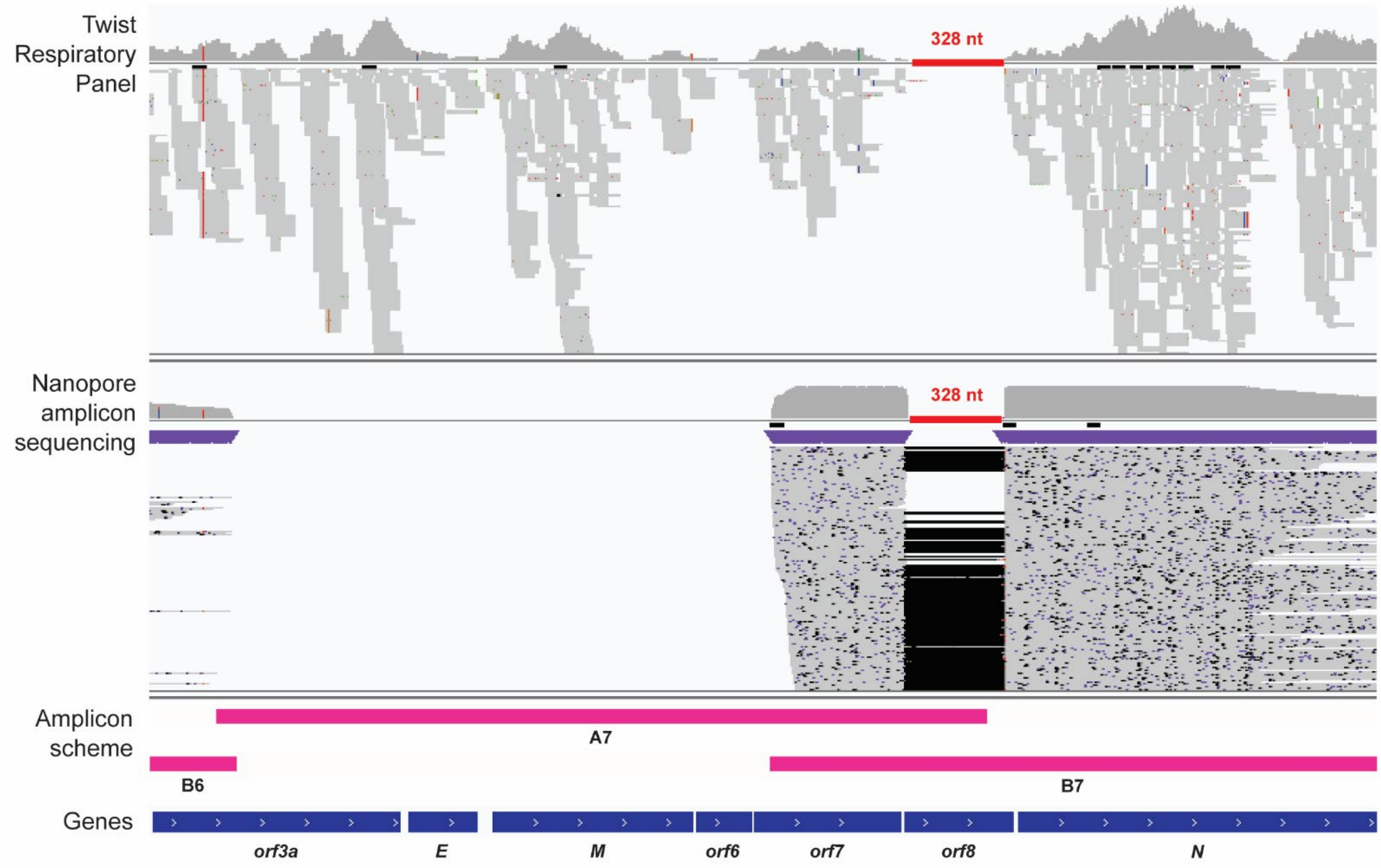

Figure S6. ***ORF8*** **deletion detected by Twist enrichment sequencing and amplicon WGS.** Genome browser view of Illumina (upper) and ONT (lower) sequencing reads aligned across the SARS-CoV-2 genome of nCoV_225 case specimen, zoomed in at the site of 328 nt *ORF8* deletion (red). ONT sequence alignment shows loss of coverage in the region targeted by the A7 amplicon primers, due to the deletion of a primer-binding site within *ORF8*. In contrast, Twist sequence reads align across this region, providing greater breadth of coverage of the reference genome (MN908947.3).
